# Cytotoxicity against Human Hepatocellular Carcinoma (HepG2) Cells and Anti-Oxidant Activity of Selected Endemic or Medicinal Plants in Sri Lanka

**DOI:** 10.1155/2022/6407688

**Published:** 2022-03-30

**Authors:** Jeyaraj Thusyanthan, Nimesha Sulochani Wickramaratne, Kanishka Sithira Senathilake, Umapriyatharshini Rajagopalan, Kamani Hemamala Tennekoon, Ira Thabrew, Sameera Ranganath Samarakoon

**Affiliations:** Institute of Biochemistry Molecular Biology and Biotechnology, University of Colombo, No. 90, Cumaratunga Munidasa Mawatha, Colombo-3, Sri Lanka

## Abstract

Hepatocellular carcinoma (HCC) is the most fatal cancer globally with limited treatment options. Plants and herbs have been used to treat cancer and other diseases for a long time by traditional practitioners in Sri Lanka. In the present study, leaf and bark extracts of selected plants were investigated for cytotoxic properties on HepG2 cells. Anti-oxidant activity and total phenolic and flavonoid contents were also determined. Plant extracts that exerted cytotoxic effects on the HepG2 cell line with IC_50_ <100 *μ*g/mL were tested on normal liver epithelial cells (THLE-3). Out of the 56 extracts, 21 exhibited potent cytotoxic effects (IC_50_ < 100 µg/mL) on HepG2 cells after 48 h exposure, and 12 were less toxic (IC_50_ > 100 *μ*g/mL) to THLE-3 normal liver cells. Six extracts exhibited potent radical scavenging activity with EC_50_ < 100 *μ*g/mL against 2,2-diphenyl-1-picrylhydrazyl (DPPH) assay, while 17 extracts showed potent anti-oxidant activity (Trolox equivalents > 100 mg/g) against ferric reducing anti-oxidant power (FRAP) assay. Out of the 56 extracts, 15 had total phenolic content above 100 mg/g of gallic acid equivalents, and 4 had flavonoid content above 100 mg/g of quercetin equivalents. Among the extracts screened, hexane, dichloromethane, ethyl acetate, and methanol extracts of *Allophylus cobbe* leaves (IC_50_ – 9.388, 6.8, 19.95, and 11.3 *μ*g/mL, respectively), *Madhuca longiflora* bark (IC_50_ – 14.42 *μ*g/mL), methanol extract of *Munronia pinnata* bark (IC_50_ – 52.06 *μ*g/mL), and hexane, dichloromethane, ethyl acetate, and methanol extracts of *Adenanthera bicolor* (IC_50_ – 45.86, 27.35, 24.56, and 61.83 *μ*g/mL, respectively) exerted potent cytotoxicity against HepG2 with less toxicity (IC_50_ > 100 *μ*g/mL) to THLE-3 cells after 48 h of incubation. These findings provide a direction to isolate possible anti-cancer compounds for hepatocellular carcinoma.

## 1. Introduction

Liver cancer is one of the cancers with a high mortality rate worldwide, and it is challenging to cure. The highest incidence of liver cancer is in Asia and Africa. More than 75% of liver cancer incidents were reported from Asian countries. Epidemics of liver cancers are increased around the world including India and the USA [1]. A significant number of patients were also reported in Sri Lanka in past years. Based on the National Cancer registry published in 2020, there were 229 males (2.1%) and 92 females (0.8%) reported for HCC out of 11,773 cancer patients in 2012 [2]. Hepatocellular carcinoma (HCC) is a primary cancer of the liver that is predominant in developing countries [3]. HCC is occurred by the presence of hepatocellular damage through reactive oxygen species and the generation of chronic inflammation related to hepatocarcinogenesis [4]. The human body reacts in various ways to counteract hepatocarcinogenesis. One such way is apoptosis or programmed cell death that maintains homeostasis between cell death and cell proliferation. Nuclear fragmentation and nuclear condensation are major morphological changes that occur during apoptosis. Cells that acquire morphological changes are phagocytized by macrophages. Apoptosis is mediated via either extrinsic (death receptor), intrinsic mitochondrial pathway, or intrinsic endoplasmic reticulum pathway. Therefore, selective induction of apoptosis of cancer cells is one of the targets for the treatment of cancer [5].

Traditional medical practitioners in Sri Lanka use plants and plant-based formulations to treat cancers [6]. Plants are a major source of a variety of secondary metabolites including phenolic (e.g., phenolic acids, flavonoids, quinones, coumarins, lignans, stilbenes, tannins), nitrogen-containing compounds (e.g., alkaloids, amines, betalains), vitamins, terpenoids (including carotenoids), and other endogenous metabolites [7–13]. Flavonoid and phenolic acids are important classes of secondary metabolites capable of scavenging free superoxide radicals, promoting anti-aging, and reducing the risk of cancer [14–16] and improve plant aroma, coloration, and flavor [17]. Phenolic compounds act as protective agents against invading plant pathogens [18–21]. Phenolic and flavonoids are reported to exhibit anti-ulcerative, anti-inflammatory, anti-oxidant, cytotoxic, anti-cancer, and anti-depressant activities [20]. Intake of phenolic compounds reduces the risk of degenerative diseases accompanied with oxidative stress including cardiovascular disease, diabetes, and obesity [22]. Anti-oxidant activity is a fundamental property of most of the bioactive compounds with anti-carcinogenic and anti-aging properties [23–25].

Different treatment modalities including radiation therapy, chemotherapy, and surgery are currently used in the treatment of liver cancer. However, a successful anti-liver cancer drug with no or minimum toxic effects is unavailable to date. Sri Lanka having rich biodiversity possesses plants that have not yet been systematically investigated for both their chemical composition and bioactivities. Therefore, the current study was undertaken to screen and identify potent Sri Lankan plant sources that can provide novel and effective anti-liver-cancer compounds as well as to characterize their anti-oxidant activities.

## 2. Materials and Methods

### 2.1. Chemicals and Reagents

Human hepatocellular carcinoma HepG2 (HB 8065™) and human normal liver epithelial cell line THLE-3 (CRL-11233™), Dulbecco's Modified Eagle's Medium (DMEM-Cat. No. 30-2002), fetal bovine serum (FBS-Cat. No. 30-2020), trypsin-EDTA (Cat. No. 30-2101), and penicillin and streptomycin (Cat. No. 30-2300) were purchased from American Type Culture Collection (ATCC; Manassas, VA, USA). Bronchial Epithelial Cell Growth Medium BulletKit™ (BEGM- CC-4175) and BEGM were purchased from Lonza/Clonetics Corporation, Walkersville, MD 21793 (BEGM Bullet Kit; CC3170).

Sulforhodamine B (SRB) powder (Cat. No. 230162), 1,1-diphenyl-2-picrylhydrazyl (DPPH), quercertin (Cat. No. Q0125), gallic acid (Cat. No. G7384), dimethyl sulfoxide (DMSO, D8414), trichloroacetic acid (TCA-T6399), acetic acid (A 6283), aluminium chloride (AlCl_3_), hexane (C_6_H_12_), dichloromethane (CH_2_Cl_2_), ethyl acetate (CH_3_COOC_2_H_5_), and methanol (CH_3_OH) were purchased from Sigma-Aldrich (St. Louis, MO, USA).

### 2.2. Cell Lines and Maintenance of Cell Culture

The human hepatocellular carcinoma cell line (HepG2) and the normal liver epithelial cell line (THLE-3) were maintained according to ATCC guidelines. HepG2 and THLE-3 cells were cultured in DMEM and BEGM, respectively with 0.1% streptomycin/penicillin and 10% fetal bovine serum. Both cells were incubated in a humidified incubator at 37°C under an atmosphere containing 5% CO_2_. The cells were subcultured at preconfluent densities using 0.25% trypsin-EDTA.

### 2.3. Collection and Authentication of Plant Material

Fresh and healthy leaves and bark of seven selected endemic and medicinal plants were collected from Pitigala, Kaluthara district, the western province of Sri Lanka. Plants were identified by the Botanists at the National Herbarium, Royal Botanical Garden, Peradeniya, Sri Lanka. Voucher specimens were deposited, and the details are given in Table 1.

### 2.4. Preparation of Plant Extracts

Plant leaves and barks were air-dried and ground into fine powder. Ground powder (5 g each) was sequentially extracted in hexane, dichloromethane, ethyl acetate, and methanol by sonication. The extracts were then filtered and dried in a rotary evaporator at room temperature. Stock solutions were prepared by dissolving dried extracts in appropriate amount of dimethyl sulfoxide (DMSO). The dilution series of each extract was prepared by adding DMEM containing 10% FBS.

### 2.5. Cytotoxicity Assay

Sulforhodamine B (SRB) assay was performed to determine cytotoxicity (IC_50_ value) of plant extracts against HepG2 cells as described by Samarakoon et al. [26] with some modifications. HepG2 cells were seeded into a 96-well plate (5,000 cells/well) with 200 *μ*L of DMEM containing 10% of FBS and incubated at 37°C for 24 h. Cells cultured in vitro were exposed in triplicates to each plant extract (12.5–200 *μ*g/mL) and incubated for 48 h. Thymoquinone (1.5–25 *μ*g/mL) was used as the positive control. Cells were observed under a light microscope after 48 h of incubation, and an SRB assay was performed to determine cytotoxicity. Absorbance was read using Synergy™ HT multimode microplate reader at 540 nm. Percentage of cell viability was calculated, and IC_50_ values were determined using GraphPad Prism® software (version 6.01). Extracts identified to have potent cytotoxicity (IC_50_ < 100 *μ*g/mL) on HepG2 cells were also tested on the normal liver epithelial cell line (THLE-3), and cytotoxicity was determined after 48 h of incubation.

### 2.6. Detection of Morphological Changes by Light Microscopy

HepG2 cells were exposed to different concentrations (12.5–200 *μ*g/mL) of each plant extract for 48 h. After the exposure period, cells were observed under an inverted light microscope (Olympus CKX41SF, Japan) to detect any morphological changes related to apoptosis.

### 2.7. 2,2-Diphenyl-1-Picrylhydrazyl (DPPH) Assay

2,2-diphenyl-1-picrylhydrazyl (DPPH) assay was used to determine radical scavenging activity as described by Ediriweera et al. with some modifications [27]. Dilution series (7.8125–1,000 *μ*g/mL) of each plant's leaf and bark extracts were prepared using dimethylsulfoxide (DMSO) in triplicates. Sample (50 *μ*L), DPPH dye reagent (90 *μ*L of 2 mg/mL), and DMSO (60 *μ*L) were added, mixed well, and incubated in the dark for 30 min. Absorbance was measured at 517.0 nm with Synergy™ HT multimode microplate reader. Percentage of inhibition was calculated according to the following equation:

(1) $$\begin{array}{c} \%\text{ inhibition }=\;\frac{\text{ABS}\left(\text{control}\right)-\text{ABS}\left(\text{sample}\right)}{\text{ABS}\left(\text{control}\right)}\;\times \;100. \end{array}$$

where ABS_(control)_ = absorbance of control and ABS_(sample)_ = absorbance of sample. The effective concentration of sample required to scavenge 50% (EC_50_) was determined by linear regression analysis of the curve plotted between percentage inhibitions versus concentration. Ascorbic acid was used as a positive control. Extracts having EC_50_ < 100 *μ*g/mL were considered as having considerable anti-oxidant activity.

### 2.8. Ferric Reducing Anti-Oxidant Power (FRAP) Assay

Ferric reducing anti-oxidant power (FRAP) was used to determine the anti-oxidant activity of plant extracts as described by Benzie and Strain [28]. Dilution series (7.825–1,000 *μ*g/mL) was prepared using acetate buffer (pH 3.6). Sample (20 *μ*L) acetate buffer (70 *μ*L) and FeCl_3_ solution (60 *μ*L) were mixed to take a preabsorbance reading. Prepared 50 *μ*L of FRAP reagent (acetate buffer:FeCl_3_:2,4,6-tri-pyridyl-s-triazine (TPTZ) as 10:1:1) was added, mixed, and incubated at room temperature for 15 min, and the absorbance was measured at 600 nm using acetate buffer as the blank. Trolox was used as the positive control. Anti-oxidant activity was determined as w/w of Trolox equivalents (TE in mg/g). Extracts having Trolox equivalents greater than 100 mg/g were considered as having potent anti-oxidant activity by FRAP assay.

### 2.9. Total Phenolic Content

The total phenolic content of the plant extracts was determined by the Folin–Ciocalteu method described by Ediriweera et al. with some modifications [27]. Dilution series (7.825–1,000 *μ*g/mL) was prepared using distilled water in triplicates. Sample (20 *μ*L) and Folin–Ciocalteu's reagent (110 *μ*L) were mixed, and the initial absorbance reading was measured at 765 nm using distilled water as the blank. A total of 10% of Na_2_CO_3_ solution (70 *μ*L) was added, mixed, and incubated for half an hour, and the final absorbance was determined. Gallic acid was used as the positive control. The phenolic content of the test samples was calculated and expressed as w/w dry weight of gallic acid equivalents (GAE in mg/g).

### 2.10. Total Flavonoid Content

The total flavonoid content of the plant extracts was determined by a modified Dowd method as described by Ediriweera et al. [27]. Dilution series (7.825–1,000 *μ*g/mL) of extracts was prepared in methanol. The initial absorbance was recorded at 415 nm using Synergy™ HT multimode microplate reader. Freshly prepared AlCl_3_ solution (100 *μ*L) was added to 100 *μ*L to each dilution and mixed thoroughly. The reaction mixture was mixed well and incubated for 10 minutes at room temperature, and final absorbance reading was taken at 415 nm using methanol as the blank. Quantification was done using a standard curve for quercetin. The results were expressed in quercetin equivalents (QE in mg/g).

### 2.11. Statistical Analysis

Statistical analysis was performed with GraphPad Prism® software version 6.01 (GraphPad Software Inc., San Diego, CA, USA). The results of anti-oxidant activity, total phenolic content, and total flavonoid content were expressed as mean ± standard deviation of three independent experiments. One-way analysis of variance (one-way ANOVA) was used to compare multiple data. The Pearson correlation coefficient was used to calculate the correlation between two variables, while statistical significance was determined by the two-tailed *t*-test. It was considered that *p* < 0.05 was statistically significant [29].

## 3. Results and Discussion

Medicinal plants play a major role as therapeutic agents in traditional medicine in developing countries [16]. In the present study, selected endemic and/or medicinal plants that are used in traditional medicine in Sri Lanka were screened for cytotoxicity. Fifty-six extracts that included hexane, dichloromethane, ethyl acetate, and methanol extracts from the leaves and bark of seven endemic and/or medicinal plants were assessed for anti-cancer activity using HepG2 cells *in vitro* (Table 2). The normal liver epithelial cell line (THLE-3) was used as the representative of normal human cells for assessing the toxicity of the plant extracts.

Among the extracts tested, dichloromethane extract of *Adenanthera bicolor* bark; hexane, dichloromethane, ethyl acetate, and methanol extracts of *Allophylus cobbe* leaves; methanol extract of *Munronia pinnata* bark; ethyl acetate and methanol extracts of *M. pinnata* leaves; hexane, dichloromethane, ethyl acetate, and methanol extracts of *Schumacheria castaneifolia* bark; and hexane, dichloromethane, and ethyl acetate extracts of *S. castaneifolia* leaves exerted potent cytotoxicity (IC_50_ < 100 *μ*g/mL) against HepG2 cells (Table 2).

**Figure 1:**
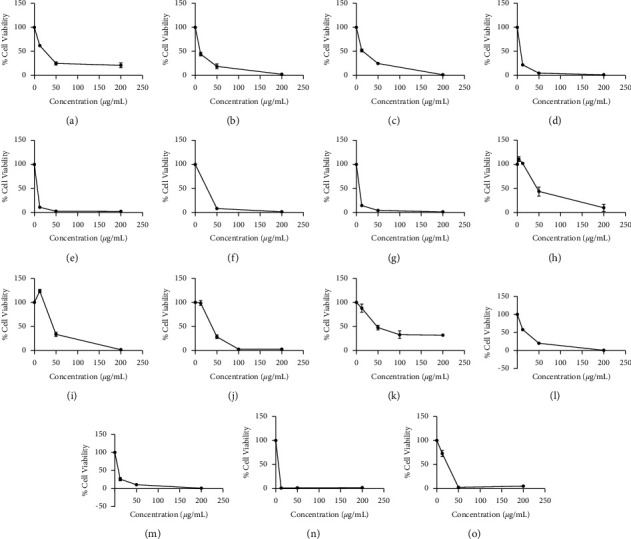
Cytotoxicity of selected active plant extracts against HepG2 cells after 48 h of incubation (dose concentration range: 12.5‐200 *μ*g/mL) (a) *A. cobbe* leaves - hexane extract, (b) *A. cobbe* leaves - dichloromethane extract, (c) *A. cobbe* leaves ‐ ethyl acetate extract, (d) *A. cobbe* leaves - methanol extract, (e) *A. bicolor* leaves - hexane extract, (f) *A. bicolor* leaves - dichloromethane extract, (g) *A. bicolor* leaves - ethyl acetate extract, (h) *A. bicolor bark* ‐ dichloromethane extract, (i) *M. pinnata* leaves - ethyl acetate extract, *S. castaneifolia* leaves extracts, (j) dichloromethane, and (k) ethyl acetate and S. castaneifolia bark extracts (l) hexane, (m) dichloromethane, (n) ethyl acetate, and (o) methanol extracts.

**Figure 2:**
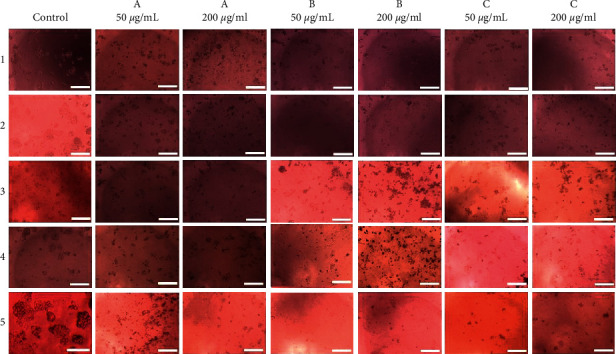
Microscopic observations of HepG2 cells (magnification = ×100) treated with selected active plant extracts (IC_50_ < 50 *μ*g/mL) after 48 h incubation; 50 *μ*g/mL and 200 *μ*g/mL of (1A) *A. cobbe* leaves – hexane, (1B) *A. cobbe* leaves – dichloromethane, and (1C) *A. cobbe* leaves – ethyl acetate; (2A) *A. cobbe* – methanol, (2B) *A. bicolor* leaves – hexane, and (2C) *A. bicolor* leaves – dichloromethane; (3A) *A. bocolor* leaves – ethyl acetate, (3B) *A. bicolor* bark – dichloromethane, and (3C) *M. pinnata* leaves – ethyl acetate; *S. castaneifolia* leaves extracts (4A) dichloromethane and (4B) ethyl acetate and *S. castaneifolia* barks (4C) hexane; and *S. castaneifolia* barks (5A) dichloromethane, (5B) ethyl acetate, and (5C) methanol. Scale bar 400 *μ*m.

All the extracts of *A. cobbe* leaves (hexane, dichloromethane, ethyl acetate, and methanol) were highly cytotoxic against HepG2 with low IC_50_ values (<20.0 *μ*g/mL) and showed less cytotoxicity (150 < IC_50_ < 300 *μ*g/mL) to THLE-3 cells. Dichloromethane extract of *A. cobbe* leaves showed the highest cytotoxic effects (IC_50_ = 6.8 *μ*g/mL) among all the extracts. Thus, the extracts of *A. cobbe* leaves appear to contain potential cytotoxic compounds that target liver cancer cells. Hexane, dichloromethane, and ethyl acetate extracts of *A. cobbe* exhibited less anti-oxidant activity with EC_50_ > 100 *μ*g/mL and TE < 15 mg/g in DPPH and FRAP assays, respectively, along with the very low quantity of total phenolics (GAE < 10 mg/g). Methanol extract *A. cobbe* leaves showed the best anti-oxidant activity (EC_50_ < 100 *μ*g/mL and TE > 150 mg/g) with significance correlation (*p* < 0.05). Total phenolic and total flavonoid contents of methanol extract of *A. cobbe* leaves are comparatively higher than that of other extracts of *A. cobbe* leaves (80–100 mg/g of GAE and 30–40 mg/g of QE, respectively) with no correlation to anti-oxidant activity (*p* > 0.05). Previous investigators have reported a lower cytotoxic (IC_50_) activity for a water extract of *A. cobbe* leaves (431.10 ± 15.05 *μ*g/mL for DU-145 and 362.08 ± 24.17 *μ*g/mL for PC-3 cell lines at 72 h) though it had a higher total phenolic content (91.96 ± 0.61 mg/g GAE) [30]. The viability of HepG2 cells decreased with increasing concentrations of hexane, dichloromethane, ethyl acetate, and methanol extracts of *A. cobbe* leaves after 48 h of exposure (Figures 1(a)–1(d)), and the characteristic apoptotic changes including shrunken cells and reduced cell volume (compared to the control) were observed in cells treated with these extracts after 48 h exposure (1A, 1B, 1C, and 2A from Figure 2).

In contrast, none of *A. cobbe* bark extracts tested exhibited significant cytotoxicity against HepG2 cells at 48 h postincubation (IC_50_ > 250 *μ*g/mL). Methanol extract of *A. cobbe* bark extract exhibited better anti-oxidant activity (EC_50_ < 60 *μ*g/mL and TE > 300 mg/g) with significant correlation (*p* < 0.05) and contained a considerable quantity of total phenolic (82.43 ± 8.60 mg/g GAE) without significant correlation (*p* > 0.05). Other extracts excluding methanol extract of *A. cobbe* bark had low anti-oxidant activity (EC_50_ > 900 *μ*g/mL and TE < 60 mg/g) along with low content of total phenolics (<20 mg/g GAE) and total flavonoids (<20 mg/g QE).

An endemic plant, *A. bicolor* leaves exhibited potent cytotoxicity (IC_50_ < 60 *μ*g/mL) for all four extracts but did not possess potent anti-oxidant activity (EC_50_ > 200 *μ*g/mL). A steep drop in percentage cell viability was observed in HepG2 cells exposed to increasing concentrations of hexane, dichloromethane, and ethyl acetate extracts of *A. bicolor* leaves after 48 h (Figures 1(e)–1(g)). The curve was flattened to approximately zero level from the concentration of 50 *μ*g/mL in the above three extracts. Reduced number of cells and constrained cells were visualized through the light microscope (2B, 2C, and 3A of Figure 2). Morphological changes in treated cells indicated that the hexane and dichloromethane extracts of *A. bicolor* leaves are more cytotoxic to HepG2 cells than the ethyl acetate extract when exposed for 48 h. Methanol extract of *A. bicolor* leaves exhibited better anti-oxidant activity for FRAP assay (approximately 195 mg/g TE). All the other extracts of *A. bicolor* leaves exhibited less anti-oxidant activity for FRAP assay (<5 mg/g TE) with a very low amount of total phenolics (<3 mg/g GAE) and total flavonoids (<8 mg/g QE). Results suggest that *A. bicolor* leaves may contain phenolic compound/s with anti-oxidant activity and cytotoxic activity. Dichloromethane extract of bark exhibited cytotoxicity (IC_50_ < 50 *μ*g/mL) against HepG2 cells after 48 h exposure. However, it was also toxic to normal hepatocytes (THLE-3) with an IC_50_ of 69.8 *μ*g/mL. A high percentage of dead cells in the wells (3B of Figure 2) and rapid decrease in percentage cell viability (Figure 1(h)) provide strong evidence for the cytotoxicity of dichloromethane extract of *A. bicolor* bark to HepG2 cells at 48 h. This extract did not exhibit anti-oxidant activity (EC_50_ > 300 *μ*g/mL and TE < 5 mg/g) and had low total phenolic (<10 mg/g GAE) and total flavonoid content bark (<10 mg/g QE). Methanol extract of *A. bicolor* bark had better anti-oxidant activity (>2,000 *μ*g/mL and <40 mg/g TE) with a good quantity of total phenols (>300 mg/g GAE) with significance correlation (*p* < 0.05); however, it was not cytotoxic (IC_50_ > 1,000) to HepG2 cells. There was no significant correlation between the anti-oxidant activity resulted from DPPH and FRAP (0.1 < *p* < 1). There are no previous data on cytotoxic and anti-oxidant activity of *A. bicolor* since it is an endemic plant. However, anti-oxidant activity as well as cytotoxicity (against NCI-H460, U251, and MCF7) has been reported for *A. pavonina*, a different plant of the *Adenanthera* genus [31].

*Madhuca longiflora* bark extract (methanol), *M. pinnata* leaf extracts (ethyl acetate and methanol), *M. pinnata* bark extract (methanol), *S. castaneifolia* leaf extracts (hexane, dichloromethane, and ethyl acetate), and *S. castaneifolia* bark extracts (hexane, dichloromethane, ethyl acetate, and methanol) also exhibited potent cytotoxicity against HepG2 cells. The decreased cell volume (compared to control) and morphological appearance of shrunken cells provide evidence for apoptosis in the cells treated with these extracts (3C to 5C of Figure 2). In addition to these morphological changes, it is clear that the percentage of cell viability decreased with the increasing concentrations of the identified extracts (Figures 1(i)–1(o)). However, leaf extract of *M. pinnata* and leaf and bark extracts of *S. castaneifolia* were also toxic to the normal liver cell line (THLE-3).

Among the extracts studied, methanol extract of leaves and bark of *A. cobbe*, leaves and bark of *M. longiflora*, bark of *A. bicolor*, and leaves *of S. castaneifolia* had better anti-oxidant activity according to DPPH assay (EC_50_ value < 100.0 *μ*g/mL) and FRAP assay (>100.0 mg/g of TE) with significance correlation (*p* < 0.05) and relatively high amount of total phenolic content (>50.0 mg/g of GAE). Oxidants tend to damage DNA and lead to carcinogenesis and uncontrolled cell division. Even though these extracts have strong anti-oxidant properties, they did not exhibit significant cytotoxicity (IC_50_ value > 100.0 *μ*g/mL) except methanol extract of *M. longiflora*. Methanol extracts of *A. cobbe* leaves and *M. longiflora* bark showed potent cytotoxicity to HepG2 cells with less toxicity to normal cells (IC_50:_ HepG2 – 11.3 and 54.42 *μ*g/mL vs. THLE-3 – 269.9 and 101.7 *μ*g/mL, respectively) and had potent anti-oxidant activity and a considerable quantity of total phenolics. Dichloromethane and ethyl acetate extracts of *A. cobbe* leaves were cytotoxic to HepG2 cells with less toxic to THLE-3 (IC_50_ > 100 *μ*g/mL) and a considerable amount of flavonoid content but anti-oxidant activity.

Hexane, dichloromethane, and ethyl acetate extracts of *S. casteneifolia* leaves exhibited potent cytotoxicity (IC_50_ as 35–60 *μ*g/mL) to HepG2 cells after 48 h of incubation. They were toxic to normal liver cells (IC_50_: 74.61, 12.35, and 14.88 *μ*g/mL for hexane, dichloromethane, and ethyl acetate extracts, respectively, after 48 h exposure). Anti-oxidant activity of these three extracts were considerably low (EC_50_ > 100 *μ*g/mL for DPPH assay and TE < 40 mg/g for FRAP assay). Hexane extract of *S. casteneifolia* leaves contained a good quantity of total phenolics (210.24 ± 12.31 mg/g GAE), while dichloromethane and ethyl acetate extracts did not (GAE <10 mg/g). The total flavonoid content of these three extracts were below 20 mg/g of QE. Even though the methanol extract of *S. casteneifolia* leaves exhibited less cytotoxicity (IC_50_ > 150 *μ*g/mL) to HepG2 cells, methanol extract of *S. casteneifolia* leaves exhibited better anti-oxidant activity (EC_50_ < 1 *μ*g/mL for DPPH and TE > 800 mg/g for FRAP assays) along with a good quantity of total phenolics (210–230 mg/g of GAE) and a significant amount of total flavonoids (20.14 ± 3.39 mg/g of QE). All extracts (i.e., hexane, dichloromethane, ethyl acetate, and methanol) of *S. casteneifolia* bark exhibited cytotoxicity to both HepG2 (IC_50_: <35 *μ*g/mL) and normal THLE-3 cells (IC_50_: 25–55 *μ*g/mL). Hexane and dichloromethane extracts of *S. casteneifolia* bark had low anti-oxidant activity (EC_50_ > 300 *μ*g/mL for DPPH and TE < 30 mg/g for FRAP assays) along with the low quantity of total phenols (<5 mg/g GAE) and total flavonoids (<20 mg/g QE). Ethyl acetate extract of *S. casteneifolia* bark exhibited low anti-oxidant activity (EC_50_:108.80 ± 11.69 *μ*g/mL and TE > 100 mg/g) with a good quantity of total phenols (>300 mg/g GAE) but a very low amount of total flavonoids (<5 mg/g QE). The methanol extract of *S. casteneifolia* bark on the other hand exhibited better anti-oxidant activity by FRAP assay (>1,000 mg/g TE) with a higher content of total phenolic (>300 mg/g GAE).

Although *Cyclea peltata* has been used in traditional medicine in Sri Lanka, none of the extracts from leaves or bark were cytotoxic to HepG2 cells after 48 h of treatment. Only the methanol extract of *C. peltata* had some anti-oxidant activity (TE > 150 mg/g in FRAP assay). All the extracts of leaves and barks of *Carallia brachiata* were less cytotoxic to HepG2 cells (IC_50_ > 100 *μ*g/mL) but had potent anti-oxidant activity. The methanol extract of *C. brachiata* leaves and ethyl acetate and methanol extracts of *C. brachiate* barks had some anti-oxidant activity (TE > 100 mg/g in FRAP assay). Of these, the methanol extract of *C. brachiata* leaves had a higher content of total phenols (>100 mg/g GAE), and the rest of the extracts had a lower content of phenolics and total flavonoids (<50 mg/g of GAE and <50 mg/g of TE, respectively).

Among the extracts of *M. pinnata* leaves and bark tested, ethyl acetate and methanol extracts of leaves and methanol extract of bark produced better cytotoxic effects (IC_50_ < 60 *μ*g/mL) on HepG2 cells. Unfortunately, the leaf methanol extract was also toxic (IC_50_ 28.79 *μ*g/mL) against THLE-3 cells. All the extracts of *M. pinnata* had low anti-oxidant activity for DPPH assay (EC_50_ > 300 *μ*g/mL) and a low content of total phenolics (GAE <30 mg/g). Exceptionally dichloromethane extract of *M. pinnata* had a higher quantity of total flavonoids (>100 mg/g of QE), while all the other extracts of *M. pinnata* bark had lower flavonoid content (<10 mg/g of QE). Hexane extract of *M. pinnata* bark showed good anti-oxidant activity only in FRAP assay as TE > 100 mg/g.

Methanol extract of *A. cobbe* bark, *M. longiflora* leaves, *A. bicolor* bark, and *S. casteneifolia* leaves had higher total phenolic content and potent anti-oxidant activity with less cytotoxicity to HepG2 cells and very lower total flavonoid content.

Overall results of the current study suggest that leaves and bark of *A. cobbe* demonstrated better anti-cancer activity on HepG2 cells with less toxic effects to normal cells. Although several biological functions were previously reported for *A. cobbe* and other *Allophylus* species, none of the studies have reported on their anti-cancer properties [32].

## 4. Conclusion

In the present study, all the extracts of *A. cobbe* leaves exhibited potent anti-cancer effects on the hepatocarcinoma cell line with noticeably less toxic effects on normal liver epithelial cells. No relationship was found between cytotoxicity, anti-oxidant activity, and total phenolic and flavonoid contents. It suggests that the anti-cancer activity of *A. cobbe* leaves is mediated by mechanisms other than anti-oxidant activity. Further studies are needed to isolate active compounds from plants identified to have potent cytotoxic effects and to unravel mechanisms of anti-cancer activity.

## Figures and Tables

**Table 1:** Scientific name, family name, and its usage for treatment/s of selected endemic and/medicinal plants.

Scientific name of the plant	Family name	Endemic/native	Used to treat
*Allophylus cobbe*	*Sapindaceae*	Native	Fracture
*Madhuca longiflora*	*Sapotaceae*	Native	Urticaria, fracture, snakebite, rheumatism, skin disease
*Adenanthera bicolor*	*Fabaceae*	Endemic	
*Cyclea peltata*	*Menispermaceae*	Native	Gastritis, bleeding from fresh wounds
*Carallia brachiata*	*Rhizophoraceae*	Native	Pruritus, oral ulcer, throat inflammation, stomatitis, wounds
*Munronia pinnata*	*Meliaceae*	Native	Fever, dysentery, general pain, swelling, haemorrhoids, cough, asthma, malaria, vomiting, blood disorder
*Schumacheria castaneifolia*	*Dilleniaceae*	Endemic	

**Table 2:** Cytotoxicity, anti-oxidant activity, total phenolic content, and total flavonoid content of different plant extracts. Cytotoxicity was determined by SRB assay (results are given as IC_50_ value (*μ*g/mL)). Anti-oxidant activity was determined by DPPH and FRAP assays, and results are expressed as EC_50_ (*μ*g/mL) and Trolox equivalents (mg/g), respectively. The total phenolic content and total flavonoid content were determined as mg/g of gallic acid equivalents and mg/g of quercetin equivalents, respectively.

Plant	Part	Extract	IC_50_ value (*μ*g/mL) and selectivity index (given in brackets)		DPPH assay	FRAP assay	Total phenols	Total flavonoids
					EC_50_ value (*μ*g/mL)	Trolox equivalents (mg/g)	Gallic acid equivalents (mg/g)	Quercetin equivalents (mg/g)
			HepG2	THLE-3				
*Allophylus cobbe*	Leaves	Hexane	19.46	158.1	>1,000	11.38 ± 1.41	5.06 ± 1.72	3.52 ± 2.02
		Dichloromethane	6.8	289.8	232.22 ± 19.53	3.96 ± 3.80	5.23 ± 4.00	123.42 ± 3.93
		Ethyl acetate	19.95	273.9	386.03 ±22.04	12.63 ± 5.56	7.93 ± 0.06	84.24 ± 6.35
		Methanol	11.3	269.9	72.78 ± 21.12	174.15 ± 22.87	89.26 ± 6.90	36.78 ± 2.33
	Bark	Hexane	288	—	907.30 ± 23.54	17.65 ± 8.56	2.09 ± 1.87	2.77 ± 0.15
		Dichloromethane	469.3	—	>1,000	51.14 ± 1.61	9.43 ± 4.13	9.64 ± 1.19
		Ethyl acetate	>1,000	—	>1,000	41.65 ± 1.76	15.98 ± 3.16	3.16 ± 8.98
		Methanol	352.4	—	49.58 ± 6.68	313.63 ± 35.31	82.43 ± 8.60	8.6 ± 3.5

*Madhuca longiflora*	Leaves	Hexane	>1,000	—	>1,000	6.59 ± 6.37	0.21 ± 0.02	4.42 ± 0.44
		Dichloromethane	>1,000	—	>1,000	38.64 ± 5.17	17.23 ± 0.52	99.93 ± 9.82
		Ethyl acetate	243.8	—	164.10 ± 13.28	146.77 ± 28.68	202.74 ± 5.52	38.51 ± 4.47
		Methanol	>1,000	—	0.02 ± 0.01	1,210.28 ± 344.43	660.77 ± 17.35	21.56 ± 3.23
	Bark	Hexane	99.49	296.8	>1,000	2.09 ± 0.60	0.43 ± 0.03	0.37 ± 0.09
		Dichloromethane	>1,000	—	>1,000	15.23 ± 6.11	5.24 ± 0.54	3.16 ± 1.88
		Ethyl acetate	364.8	—	158.22 ± 13.97	158.62 ± 5.76	16.46 ± 3.80	8.60 ± 5.28
		Methanol	54.42	101.7	43.26 ± 15.79	1,808.63 ± 159.63	282.7 ± 23.01	1.18 ± 0.01

*Adenanthera bicolor*	Leaves	Hexane	45.86	261.1	580.58 ± 23.50	2.65 ± 1.83	0.56 ± 0.01	0.81 ± 0.02
		Dichloromethane	27.35	182.8	968.30 ± 18.37	0.94 ± 1.14	1.65 ± 0.59	4.67 ± 1.64
		Ethyl acetate	24.56	309.1	>1,000	1.91 ± 1.21	1.55 ± 0.04	6.91 ± 2.32
		Methanol	61.83	424.5	212.74 ± 7.93	195.48 ± 23.73	52.95 ± 3.18	3.39 ± 1.79
	Bark	Hexane	103.7	>1,000	381.91 ± 57.67	4.55 ± 1.51	0.29 ± 0.05	0.05 ± 0.01
		Dichloromethane	45.87	69.8	315.64 ± 11.25	4.83 ± 0.52	5.45 ± 2.29	3.83 ± 1.23
		Ethyl acetate	236	—	152.13 ± 14.89	121.81 ± 16.31	103.40 ± 7.24	4.01 ± 3.50
		Methanol	>1,000	—	36.65 ± 1.03	2,011.32 ± 298.18	345.48 ± 12.92	0.53 ± 0.56

*Cyclea peltata*	Leaves	Hexane	>1,000	—	>1,000	3.91 ± 4.15	2.01 ± 0.94	6.78 ± 3.25
		Dichloromethane	>1,000	—	>1,000	25.55 ± 2.22	0.98 ± 0.88	4.64 ± 3.38
		Ethyl acetate	113.5	—	>1,000	7.38 ± 5.82	2.93 ± 0.73	1.82 ± 0.83
		Methanol	>1,000	—	476.34 ± 13.34	14.97 ± 4.60	15.20 ± 0.19	2.86 ± 3.11
	Bark	Hexane	>1,000	—	>1,000	3.41 ± 0.64	8.93 ± 2.04	15.57 ± 5.62
		Dichloromethane	>1,000	—	765.26 ± 75.95	3.09 ± 0.89	7.91 ± 0.46	1.41 ± 1.00
		Ethyl acetate	>1,000	—	>1,000	7.64 ± 3.41	52.93 ± 0.10	0.55 ± 0.39
		Methanol	>1,000	—	182.57 ± 25.57	171.43 ± 35.46	5.99 ± 1.68	2.22 ± 1.30

*Munronia pinnata*	Leaves	Hexane	237.6	—	>1,000	38.24 ± 9.94	1.27 ± 0.48	0.56 ± 0.4
		Dichloromethane	269.9	—	>1,000	31.73 ± 11.63	14.84 ± 2.56	142.32 ± 14.05
		Ethyl acetate	47.07	63.9	901.54 ± 28.36	6.42 ± 3.42	5.32 ± 2.23	7.88 ± 0.89
		Methanol	56.92	28.79	328.02 ± 58.92	9.10 ± 4.31	9.88 ± 0.64	2.70 ± 0.22
	Bark	Hexane	>1,000	—	>1,000	109.72 ± 12.52	2.11 ± 0.91	5.81 ± 4.36
		Dichloromethane	385.4	—	604.89 ± 62.62	13.29 ± 1.90	5.98 ± 2.33	8.85 ± 3.60
		Ethyl acetate	656	—	>1,000	11.79 ± 5.78	4.32 ± 1.55	1.40 ± 0.99
		Methanol	52.06	139.7	338.17 ± 35.61	64.55 ± 8.53	22.93 ± 1.12	10.49 ± 4.64
*Schumacheria castaneifolia*	Leaves	Hexane	58.42	74.61	491.07 ± 58.32	5.87 ± 7.86	210.24 ± 12.31	0.97 ± 0.01
		Dichloromethane	39.12	12.35	>1,000	21.39 ± 8.40	1.82 ± 0.54	13.00 ± 1.86
		Ethyl acetate	44.5	14.88	288.63 ± 6.41	39.75 ± 5.33	8.26 ± 0.40	12.08 ± 0.46
		Methanol	198.6	—	0.01 ± 0.01	836.94 ± 164.78	216.61 ± 11.6	20.14 ± 3.39
	Bark	Hexane	32.33	47.32	893.83 ± 76.26	29.31 ± 15.58	1.00 ± 0.10	19.43 ± 3.22
		Dichloromethane	6.492	50.5	331.20 ± 27.25	37.14 ± 8.46	3.87 ± 1.50	18.54 ± 6.66
		Ethyl acetate	<0.01	41.7	390.77 ± 8.09	155.18 ± 29.06	1.45 ± 0.58	11.86 ± 1.00
		Methanol	34.14	29.5	108.80 ± 11.69	1,574.97 ± 199.41	362.18 ± 11.94	3.37 ± 0.64

*Carallia brachiata*	Leaves	Hexane	668.3	—	>1,000	3.10 ± 0.30	2.00 ± 0.20	6.81 ± 2.43
		Dichloromethane	>1,000	—	>1,000	4.40 ± 0.43	1.00 ± 0.06	3.50 ± 2.00
		Ethyl acetate	>1,000	—	857.26 ± 11.89	3.91 ± 4.15	15.88 ± 1.77	16.73 ± 2.17
		Methanol	228.7	—	101.35 ± 6.77	821.05 ± 135.45	138.59 ± 6.35	10.65 ± 0.70
	Bark	Hexane	>1,000	—	>1,000	6.37 ± 2.60	6.90 ± 2.26	2.07 ± 1.89
		Dichloromethane	>1,000	—	244.60 ± 19.58	25.31 ± 6.15	46.05 ± 9.42	7.31 ± 1.03
		Ethyl acetate	174.3	—	163.33 ± 6.59	115.19 ± 18.51	11.58 ± 0.82	4.27 ± 2.13
		Methanol	101.5	—	121.59 ± 5.50	593.52 ± 88.73	2.02 ± 0.54	2.18 ± 1.59

## Data Availability

The data set supporting this article is included in the article.
